# Studying loneliness and its impact on mental health among University students: the SONDEU Project

**DOI:** 10.1192/j.eurpsy.2025.693

**Published:** 2025-08-26

**Authors:** A. Calvo, E. Lara

**Affiliations:** Personality, Assesment and Clinical Psychology Department, Universidad Complutense de Madrid, Madrid, Spain

## Abstract

**Introduction:**

The challenge of loneliness underlines the need for studies that allow us to analyse and understand this phenomenon, particularly in vulnerable groups. Although loneliness can occur at any stage of life, it seems to be more prevalent during phases of transition, where changes in social relationships are frequent (Mund et al., 2020). In the younger population, a significant restructuring of social life is observed, with peer relationships becoming more central while family ties become less prominent. This often coincides with key life transitions such as change of residence, first romantic relationships, work experiences, etc. (von Soest et al., 2020). This is especially noticeable during the university stage, a period often accompanied by academic pressure due to personal, familial or societal expectations and/or needs. Furthermore, this stage is especially critical from a mental health perspective, as most mental health problems appear for the first time before the age of 25.

**Objectives:**

1) To analyse the impact that loneliness has on the student community at the Complutense University of Madrid (UCM). 2) To find out the prevalence and types of loneliness as well as its association with different problems of mental health.

**Methods:**

This study follows a two-phase mixed-methods approach. In the first phase, two focus groups were conducted to explore the definition and characteristics of loneliness in-depth. The convenience sample consisted of 8 to 10 students per focus group from different educational levels and academic disciplines. A pre-designed guide with open-ended questions facilitated the session (e.g., “Reflecting on your university experience, what situations or experiences have made you feel lonely?”). In the second phase, a cross-sectional observational study was conducted. While we estimated a sample size of 246 participants to explore the prevalence of loneliness, we aim to obtain a representative sample of students from the UCM. The following measures were included: loneliness (frequency, type), mental health (depression, anxiety, psychotic experiences, suicidal behaviour), well-being and social relationships, academic performance, non-behavioural addictions and lifestyle. Data is being processed and analysed using appropriate statistical and qualitative analysis software. This study has received ethical approval from the participating institution.

**Results:**

Data from the focus groups and the online survey were compared from different educational levels and academic disciplines to explore characteristics of loneliness and mental health problems.

**Conclusions:**

This study seeks to gain a deeper understanding of loneliness among university students. These findings aim to promote changes that integrate a new form of care and promotion of mental health in this group (i.e., identification, prevention and management of loneliness) as well as to encourage further research in this area.

**Disclosure of Interest:**

None Declared

